# Supplementary material for: Motivation to access laparoscopic skills training: Results of a Canadian survey of obstetrics and gynecology residents
Source: PLoS One. 2020 Apr 2;15(4):e0230931. doi: 10.1371/journal.pone.0230931 (PMC7117757; doi:10.1371/journal.pone.0230931)
Supplement: S1 Appendix — (DOCX) [file pone.0230931.s001.docx]

**Canadian Survey of Ob/Gyn Residents’ Motivation to Access Laparoscopic Simulation Training**

What is your level of training?

PGY 2/3/4/5/Other FREE TEXT

In which residency program are you completing your training?

Memorial University

Dalhousie University

McGill University

University of Ottawa

Queen’s University

Western University

University of Toronto

McMaster University

University of Manitoba

University of Saskatchewan

University of Calgary

University of Alberta

University of British Columbia

Other

What is your age?

What is your gender?

M/F/ Prefer not to say

Do you plan on performing laparoscopic surgery regularly as a staff?

Yes/No/Undecided

Please check one of the following:

I have used laparoscopic simulation resources

I have not used laparoscopic simulation resources

Please check one of the following:

I have experience performing minimally invasive surgery

I do not have experience performing minimally invasive surgery

How many hours did you spend practicing laparoscopic simulation exercises (defined as peg transfer, intracorporeal knot tying) in the **past 12 months**? Please round to the nearest hour.

(FREE TEXT) hours in the past 12 months

During a typical surgical block in the last 12 months,

(ex. Core Gynaecology, Gynaecologic Oncology, Reproductive Endocrinology, Urogynaecology)

how many hours did you spend participating in minimally invasive surgery cases in the operating room **per week**? Please round to the nearest hour.

(FREE TEXT) hours per week

During a typical surgical block in the last 12 months, how often did you receive supervised practice at laparoscopic simulation exercises (supervision and coaching might be provided by a senior resident or staff)?

Never/Rarely/Sometimes/Often/Always

What percentage of the time that you spent practicing laparoscopic simulation exercises was supervised practice? (supervision and coaching might be provided by a senior resident or staff)?

FREE TEXT percent

Who supervised your practice? Check all that apply.

- Staff Ob/Gyn (including Urogynecologist, Gynecologic Oncologist)
- Senior Resident
- Other (Please Describe) FREE TEXT

How concerned are you about learning bad habits during independent, unsupervised practice of laparoscopic simulation exercises?

(not at all concerned, very concerned)

When you used simulation in the past how important were each of the following reasons for using simulation? Rate each of the following reasons in terms of their importance to you (0 = Not Applicable, 1=not at all important, 3=somewhat important, 5=very important):

- Skill development
- Interest in laparoscopic surgery
- Mandatory or protected time to use the simulation resources
- Practice before a case
- Requirement to use the simulation resources for a rotation
- Recommendation of an attending surgeon to use the simulation resources
- Proximity to simulation lab
- Sufficient free time to use the simulation resources
- Peer pressure to use the simulation resources

Are there any other reasons that you use laparoscopic skills simulation?

FREE TEXT

During which times do you access the laparoscopic simulation lab? (check all that apply)

- During work hours
- Post-call
- Off Duty/vacation
- Other (Please Specify) FREE TEXT

What prevents you from accessing laparoscopic simulation skills training? Include as many reasons as apply.

FREE TEXT

*[Note: For the following questions, 5 point Likert Scales will be used, numbered 1,2,3,4,5. Component Headings are not made available to study participants]*

The following questions will ask you about your perceptions of laparoscopic simulation exercises or minimally invasive surgery. Please indicate your response using the scale provided.

Perceived Task Value Items

Intrinsic Interest Value

The following questions pertain to exercises done in the *simulation* setting:

1. In general, I find performing laparoscopic simulation exercises (very boring, very interesting)
2. How much do you enjoy performing laparoscopic simulation exercises? (not very much, very much)
3. How much do you like laparoscopic simulation? (not very much, very much)
4. How satisfying do you find laparoscopic simulation? (not very satisfying, very satisfying)

The following questions pertain to the *operating room* setting:

1. In general, I find performing minimally invasive surgery (very boring, very interesting)
2. How much do you enjoy performing minimally invasive surgery? (not very much, very much)
3. How much do you like performing minimally invasive surgery? (not very much, very much)
4. How satisfying do you find performing minimally invasive surgery? (not very satisfying, very satisfying)

Attainment Value

1. How worthwhile is it to master minimally invasive surgery skills to you? (not worthwhile, very worthwhile)
2. I feel that, to me, being someone who is good at minimally invasive surgery skills is (not important, very important)
3. Being someone who is skilled at performing minimally invasive surgery cases is (not a high priority, a very high priority)
4. I feel that, to me, having strong laparoscopic surgery skills is (not essential, essential)
5. How important is it to you to be proficient at minimally invasive surgery? (not important, very important)

Extrinsic Utility Value

1. To me, proficiency in minimally invasive surgery is (not an asset to my career, a large asset to my career)
2. To me, strong minimally invasive surgical skills are (not valuable after residency, very valuable after residency)
3. How important is being proficient in minimally invasive surgery to finding desired employment after residency? (not important, very important)
4. How useful is learning minimally invasive surgery to your career goals? (not useful, very useful)
5. Being proficient at minimally invasive surgery (will not help me in the future, will be very helpful in the future)

The following questions pertain to exercises done in the *simulation* setting:

1. How useful are laparoscopic simulation exercises to performing minimally invasive surgeries? (not useful, very useful)
2. How transferable are laparoscopic simulation skills to the operating room? (Not at all transferrable, very transferrable)
3. Are laparoscopic simulation exercises helpful in improving minimally invasive surgery skills? (not at all helpful, very helpful)
4. How important are laparoscopic simulation exercises for becoming proficient at minimally invasive surgery? (not at all important, very important)
5. Performing laparoscopic simulation exercises will make me a better minimally invasive surgeon (strongly disagree, strongly agree)

Ability/Expectancy-Related

The following questions pertain to the *operating room* setting:

1. How would you rate your minimally invasive surgery skills? (much worse than other residents, much better than other residents)
2. How good at minimally invasive surgery are you? (Not at all good, very good)
3. How capable do you feel performing basic minimally invasive surgical procedures independently? (not at all capable, very capable)
4. How would you rate your minimally invasive surgery? (very poor, very strong)
5. If you were to order all of the residents in your program from the worst to the best at minimally invasive surgery, where would you put yourself? (the worst, the best)

Self-Efficacy

1. I am confident that I will be able to learn the skills required to become proficient at minimally invasive surgery (not at all confident, very confident)
2. I am confident that I can learn the skills to become good at minimally invasive surgery (not at all confident, very confident)
3. I am confident that I can learn sufficient minimally invasive skills to perform procedures safely (not at all confident, very confident)
4. I am confident that I can learn the skills necessary become a capable minimally invasive surgeon (not at all confident, very confident)
5. I am confident that I can motivate myself to learn the skills necessary to become a competent minimally invasive surgeon even when I’m busy (not at all confident, very confident)
6. I am confident that I can motivate myself to learn the skills to become good at minimally invasive surgery (not at all confident, very confident)
7. I am confident that I have the motivation to learn the skills required to perform minimally invasive surgery safely (not at all confident, very confident)
8. I am confident that I can independently perform a laparoscopic tubal ligation with Filshie clips safely (not at all confident, very confident)
9. I am confident that I can independently perform a laparoscopic ovarian cystectomy safely (not at all confident, very confident)
10. I am confident that I can independently perform a total laparoscopic hysterectomy safely (not at all confident, very confident)
11. I am confident that I can safely perform minimally invasive surgical skills on my own (not at all confident, very confident)

Perceived Task Difficulty

Task Difficulty

1. In general, how hard is performing minimally invasive surgery for you? (very easy, very hard)
2. How challenging is performing minimally invasive surgery for you? (much easier than for other residents, much more challenging than for other residents)
3. Compared to other aspects of Obstetrics and Gynaecology, how hard are minimally invasive surgery skills for you? (the easiest skill, the hardest skill)

Task Effort Cost

The following questions pertain to exercises done in the *simulation* setting:

1. Performing laparoscopic simulation exercises demands too much of my time (strongly disagree, strongly agree)
2. I have to put too much energy into performing laparoscopic simulation exercises (strongly disagree, strongly agree)
3. Performing laparoscopic simulation exercises takes up too much time (strongly disagree, strongly agree)
4. Performing laparoscopic simulation exercises is too much work (strongly disagree, strongly agree)
5. Performing laparoscopic simulation exercises requires too much effort (strongly disagree, strongly agree)

Outside Effort Cost

1. I have so many other commitments, I can’t put forth the effort needed to perform laparoscopic simulation exercises (strongly disagree, strongly agree)
2. Because of the other demands on my time, I don’t have enough time to perform laparoscopic simulation exercises (strongly disagree, strongly agree)
3. I have so many other responsibilities that I am unable to put in the effort that is necessary to perform laparoscopic simulation exercises (strongly disagree, strongly agree)
4. Because of the other things that I have to do, I don’t have time to put into performing laparoscopic simulation exercises (strongly disagree, strongly agree)

Loss of Valued Alternatives

1. I have to sacrifice too much to perform laparoscopic simulation exercises (strongly disagree, strongly agree)
2. Performing laparoscopic simulation exercises requires me to give up too many other activities (strongly disagree, strongly agree)
3. Performing laparoscopic simulation exercises causes me to miss out on too many things that I care about (strongly disagree, strongly agree)
4. I can’t spend as much time doing other things that I would like to do because of the time I spend performing laparoscopic simulation exercises (strongly disagree, strongly agree)

Emotional Cost

1. I worry too much about performing laparoscopic simulation exercises (strongly disagree, strongly agree)
2. Performing laparoscopic simulation exercises is too exhausting (strongly disagree, strongly agree)
3. Performing laparoscopic simulation exercises is emotionally draining (strongly disagree, strongly agree)
4. Performing laparoscopic simulation exercises is too frustrating (strongly disagree, strongly agree)
5. Performing laparoscopic simulation exercises is too stressful (strongly disagree, strongly agree)
6. Performing laparoscopic simulation exercises makes me feel too anxious (strongly disagree, strongly agree)

The following questions pertain to the *operating room* setting:

1. I worry too much about becoming proficient at minimally invasive surgery (strongly disagree, strongly agree)
2. Becoming proficient at minimally invasive surgery is too exhausting (strongly disagree, strongly agree)
3. Becoming proficient at minimally invasive surgery is emotionally draining (strongly disagree, strongly agree)
4. Becoming proficient at minimally invasive surgery is too frustrating (strongly disagree, strongly agree)
5. Becoming proficient at minimally invasive surgery is too stressful (strongly disagree, strongly agree)
6. Becoming proficient at minimally invasive surgery makes me feel too anxious (strongly disagree, strongly agree)
