# Supplementary material for: Motivation to access laparoscopic skills training: Results of a Canadian survey of obstetrics and gynecology residents
Source: PLoS One. 2020 Apr 2;15(4):e0230931. doi: 10.1371/journal.pone.0230931 (PMC7117757; doi:10.1371/journal.pone.0230931)
Supplement: S1 Table — (DOCX) [file pone.0230931.s002.docx]

| Reasons for Accessing Laparoscopic Simulation | Not Important (1-2)  % (n) | Somewhat Important (3) % (n) | Important or Very Important (4-5) % (n) |
| --- | --- | --- | --- |
| Skill development | 0 (0) | 12(5) | 88 (38) |
| Free time | 5 (2) | 12 (5) | 84 (36) |
| Interest in laparoscopic surgery | 5 (2) | 28 (12) | 67 (29) |
| Proximity to the skills lab | 9 (4) | 28 (12) | 63 (27) |
| Mandatory or protected time | 19 (8) | 33 (14) | 49 (21) |
| Practice before a case | 23 (10) | 35 (15) | 42 (18) |
| Recommendation of an attending surgeon | 28 (12) | 30 (13) | 42 (18) |
| Requirement for a rotation | 44 (19) | 35 (15) | 21 (9) |
| Peer pressure | 58 (25) | 28 (12) | 14 (6) |
